# Giant Condyloma Acuminata in Indonesian Females with SLE under Immunosuppressant and Steroid Therapy

**DOI:** 10.1155/2016/4710979

**Published:** 2016-10-24

**Authors:** Andhika Rachman, Nabila Hasan

**Affiliations:** ^1^Division of Hematology and Medical Oncology, Department of Internal Medicine, Faculty of Medicine, University of Indonesia-Cipto Mangunkusumo Hospital, Jakarta, Indonesia; ^2^Faculty of Medicine, University of Indonesia-Cipto Mangunkusumo Hospital, Jakarta, Indonesia

## Abstract

*Introduction.* Immunosuppressant and steroid therapy in systemic lupus erythematosus (SLE) increases the risk of human papillomavirus (HPV) infections, one of which is giant condyloma acuminata (GCA). To our knowledge, there is no report evaluating the correlation between immunosuppressive and steroid therapy in patients with SLE and the prevalence of GCA.* Case Report.* A 42-year-old female was diagnosed with SLE a year ago and has been treated with steroids and immunosuppressive drugs. In the last few months she presented GCA involving the genital area recurring almost every two months. Type 6 and 11 HPVs were identified in vulva, vagina, and cervix.* Methods.* PubMed, EBSCO, and Cochrane Library literature were searched from inception to July 2015. Authors screened all titles and abstracts and read full text article, and two case-control studies were found relevant.* Results.* SLE patients in both studies were under immunosuppressive and steroid therapy. Condyloma acuminata was diagnosed at 108 months (latest) and 1 month (earliest) after SLE. Type 6, 11, 16, 42, and oncogenic group of HPV were identified.* Conclusions.* GCA is a type of HPV infection seldom observed in SLE patients. Therefore, their correlation is still unclear. Period of time since SLE was diagnosed and GCA varies from months to years. A more thorough physical and laboratory examination leading to HPV and other infectious disease is recommended.

## 1. Introduction

Infection is an important cause of morbidity and mortality in patients with systemic lupus erythematosus (SLE), including infections in the genitalia, such as infections caused by human papillomavirus (HPV) [1]. In Indonesia, HPV 16 and 18 are equally common in the general population, as they are in cervical cancer [2].

Giant condyloma acuminata (GCA), a type of HPV infection in the genital area, is a benign tumor that grows resembling a cauliflower. Although GCA is benign, it has malignancy tendencies as it can grow up to 10 cm, which is locally invasive and damaging [3]. It is a very rare sexually transmitted infection (STI), with 0.1% incident in general population [4, 5].

SLE is a systemic autoimmune disease characterized by high level of autoantibodies affecting many internal organs and therefore, its clinical manifestation varies. The disease is mainly treated with immunosuppressant and steroid. Some studies reported that immunosuppressant and steroid therapy in SLE patient may increase the risk of developing HPV infection [6]. Santana et al. [7] and Nath et al. [8] did not find the correlation between the use of immunosuppressant and the prevalence of HPV infection. To our knowledge, there is no report in South East Asia that evaluates the correlation between immunosuppressant such as azathioprine, cyclophosphamide, mycophenolate mofetil, and steroid therapy in patients with SLE and the prevalence of giant condyloma acuminata.

## 2. Case Report

**Figure 1:**
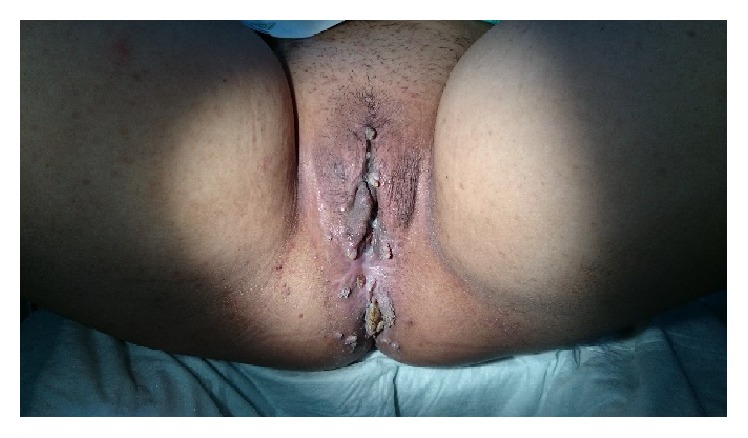
Genital warts in vulvar and anal region.

**Figure 2:**
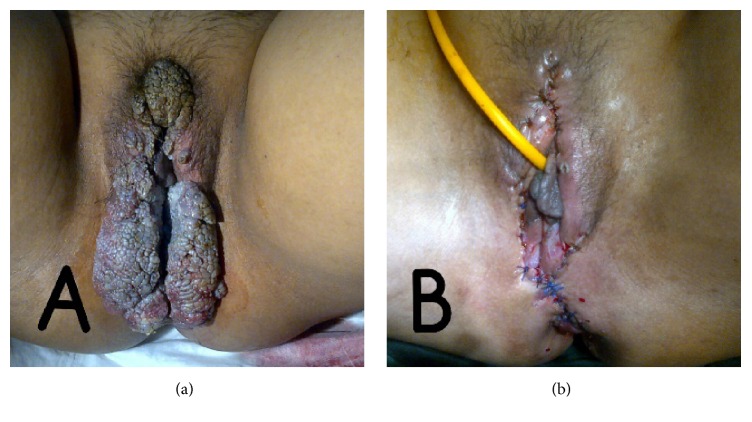
(a) Recurrence of condyloma acuminata in patient with systemic lupus erythematosus during methylprednisolone and mycophenolate mofetil therapy (b) after surgical procedure.

**Figure 3:**
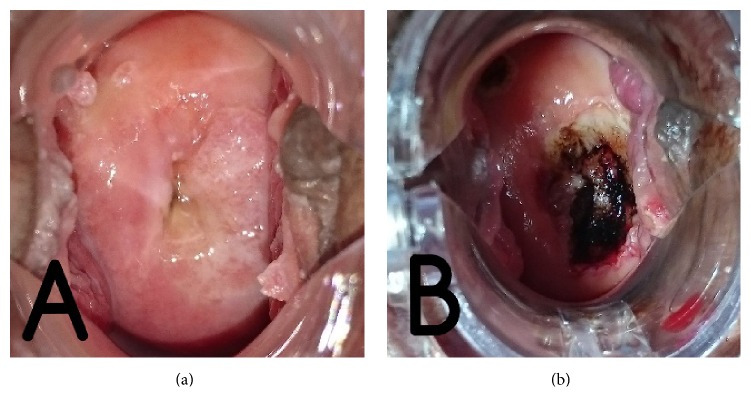
(a) Recurrence of condyloma acuminata in cervical region (b) after surgical procedure.

The patient, a 42-year-old female, was diagnosed with SLE in 2013 based on the presence of antinuclear antibodies, in addition to discoid rash and arthritis. She is sexually active with one partner, had one child, and had a history of multiple sexual partners. Since then she has been treated with methylprednisolone (32 mg/day) and mycophenolate mofetil (1000 mg/day). After six months of therapy, genital warts first appear in the vulva and anal region (Figure 1) and recurred in two months spreading to the pubic mound, perineum, and anal region (Figure 2(a)). It became progressively hypertrophic, resembling cauliflower. Biopsies were performed showing papillomatosis and hyperkeratosis. Type 6 and 11 HPV were identified in the lesions through RT-PCR. She was treated with excision (Figure 2(b)). The following month, she came with HPV infection in cervical region and was treated with surgical procedure (Figures 3(a) and 3(b)).

## 3. Clinical Question

In this report, author would like to know if immunosuppressant and steroid therapy in patient with SLE induced giant condyloma acuminate and which regimen induces it rapidly.

## 4. Methods

Electronic searches of PubMed, EBSCO, and Cochrane Library were conducted from inception to July 2015. Search terms (in English) are “SLE AND condyloma acuminatum AND immunosuppressant AND steroid”. There are only few articles found in literature search. Therefore, first article screening was done by reading all titles and abstracts. Full text articles were assessed for eligibility, and two articles were found relevant (Table 1).

## 5. Results

Only two case-control studies related with immunosuppressant and steroid therapy in SLE and the prevalence of giant condyloma acuminata were found. Both studies have low validity compared to randomized controlled trial (RCT) and systematic reviews.

In the first study, Lube et al. [9] reported the incident of GCA in childhood systemic lupus erythematosus (C-SLE). The study reported that 289 of 5682 patients in Pediatric Rheumatology Unit were diagnosed with SLE, and 4 of 289 (1.4%) had GCA. All patients were under immunosuppressant and corticosteroid therapy.

In the second study, Costapinto et al. [10] reported the incident of condyloma acuminata in a 33-year-old Black female who was diagnosed with SLE during her first pregnancy in 2003. In 2004, after seven months of mycophenolate mofetil therapy, she was diagnosed with condyloma acuminata in the genital area. The patient had used steroids and immunosuppressant such as cyclophosphamide and azathioprine since she was 14 years old due to her irregular menstrual cycle. Detailed characteristic of each patient in both studies can be seen in Table 2.

## 6. Discussion

There are not many literatures found discussing the correlation between immunosuppressant and steroid therapy in patients with SLE and the prevalence of condyloma acuminata. It may be due to its low incidence, which is only 0.1% in general population [4, 5]. Thornsberry and English III [11] stated that the incidence of anogenital warts, a symptom of GCA, is approximately 1% in sexually active adults and may be up to 3% in sexually active adolescents. HPV is known as the most common sexually transmitted disease (STD) in United States females and the major risk factor is younger age at first sexual intercourse [12], as observed in cases in both studies, where all patients are sexually active except for one case that has a history of sexual abuse.

Systemic lupus erythematosus is a disease with impaired immune regulation and response. The number of active B cells producing immunoglobulin is increasing in the peripheral blood. B cells in SLE patients are likely to undergo polyclonal B cell activation by a specific antigen. B cells responses are abnormal to signal activity. B cell hyperactivity is characterized by elevated concentrations of interleukins 6 and 10 (IL-6 and IL-10). In addition, the number of activated T cells in peripheral blood is also increasing. In SLE patients, T cells may provide help for increasing the production of antibodies. The phagocytic cells in SLE patients can not bind or process immune complex and thus stimulates the formation of antibodies [13].

NK cells play an important role in the innate immune system. They recognize and kill viruses, such as HPV, and transform cells through two mechanisms, that is, granule-dependent cytotoxicity and apoptosis in target cells. NK cells interact with HPVs and can participate in immune response against HPV-induced lesions by displaying higher cytotoxic activity and cytokine production (TNF-*α* and IFN-*γ*) [14, 15]. Meanwhile, Henriques et al. [16] found that numbers of circulating NK cells are lower in patients with SLE when compared to healthy subjects, more notorious in active disease. Thus, patients with SLE can be prone to HPV infections, such as condyloma acuminata. Patients with SLE have more severe tissue damage as a result of continuing abnormalities in immune response and regulation.

Therefore, immunosuppressant and steroid therapy is given. However, the therapy itself is one of the main risk factors for developing HPV infection, especially in the skin area (OR 2.91, 95% CI 1.18–7.14). In addition to SLE (OR 2.16, 95% CI 1.04–4.48) [13]. Mendoza-Pinto et al. [17] found that cumulative glucocorticoid dose in women with SLE may increase the risk of HPV infection. Glucocorticoid such as prednisone suppressed the functions of Toll-like receptor (TLR) stimulated plasmacytoid dendritic cells, thereby reducing the ability to clear HPV infection. Mycophenolic acid was also associated with cervical HPV infection. A Mexican study detected low levels of B and NK cells and an increased risk of HPV infection in SLE patients receiving mycophenolate mofetil [18]. Klumb et al. [19] found that there is a higher cumulative cyclophosphamide dose in SLE patients with HPV infections.

In both studies [9, 10], all patients have immunosuppressant therapy, such as azathioprine, chloroquine, hydroxychloroquine, intravenous cyclophosphamide, or mycophenolate mofetil, while steroids used are prednisone and methylprednisolone. Therapy was given immediately after SLE was diagnosed. The earliest condyloma acuminata incident was found in the third case with only one-month period after the diagnosis of SLE.

Steroids and immunosuppressants have a mechanism to inhibit B cells, T cells, and inflammatory cytokines, which are very appropriate to be used as the rationales of SLE therapy. Glucocorticoids inhibit the extravasation and infiltration of leucocytes into tissue and inhibit macrophages and lymphocytes (B and T cells) to produce inflammatory cytokines such as TNF-*α*, IL-1, IL-12, and IFN-*γ*, while immunosuppressant such as mycophenolate mofetil, which is derived from mycophenolic acid isolated from* Penicillium glaucum*, inhibits the response of lymphocytes through purine synthesis [20].

The most frequent types of HPV found in patients with SLE are types 6 and 11, which are low-risk HPVs (LR-HPVs) associated with benign tumor. In addition to types 16 and 18, there are other types of HPV that may be found and also play a role in malignancy, which are considered as high-risk HPVs (HR-HPVs) [21]. In Lube et al.'s study [9], all patients have type 16 and oncogenic group with a risk to dysplasia and cervical cancer.

Immunization can be considered as a primary prevention to HPV spread. At the end of 2008, 70% of women younger than 26 years of age had HPV vaccine and the there was a marked reduction in the incidence of condyloma acuminata (50% compared to 2004–2007) [22]. Gardasil, the currently available quadrivalent HPV vaccine, can increase the NK cell population following immunization [14]. Mok et al. [23] found in their study that the quadrivalent HPV vaccine is well tolerated and reasonably effective in patients with stable SLE and does not induce an increase in lupus activity or flares.

Both studies [9, 10] showed variety of condyloma therapy, such as the use of trichloroacetic acid, TLR7 agonist, imiquimod 5% cream, podophyllin 2% oil, CO_2_ laser vaporization, LEEP, and surgical procedure. Aubin et al. [24] distinguish condyloma therapy into three categories: physical destruction, chemical, and immunomodulation. However, no therapy can totally eradicate the disease. One of three patients in Lube et al.'s study showed recurrence [9]. Therapy given in condyloma acuminata is based on location, number, size, availability of medical instrument, and doctor's experience.

## 7. Conclusions

GCA is a type of HPV infection which is seldom observed in SLE patients. Period of time since SLE was diagnosed and GCA varies from months to years. The correlation between immunosuppressant and steroid therapy in SLE patients and the prevalence of giant condyloma acuminata is still unclear. Therefore, further study and research with bigger sample are required. Hopefully, the studies can be used as a reference in choosing therapy regimens in patients with SLE or other autoimmune diseases. A more thorough history taking, physical, and laboratory examination leading to HPV and other infectious diseases should be performed as a routine procedure in clinical practice. HPV vaccine, particularly before first sexual intercourse, is recommended in SLE patients prior to administration of immunosuppressive and steroid therapy.

## Figures and Tables

**Table 1:** Literature search.

Source	Keywords	Result
PubMed	SLE AND condyloma acuminatum AND Immunosuppressant AND Steroid	1
EBSCO		1
Cochrane Library		0

**Table 2:** Characteristic of each patient in both studies.

Variable	Lube et al. [9]				Costapinto et al. [10]
	1	2	3	4	1
*Demographic data*					
Age at SLE diagnosis, years	12	8	14	15	33
Gender	♀	♀	♀	♀	♀
SLE therapy	Chloroquine 5 mg/kg/day; prednisone 60 mg/day	Prednisone 2 mg/kg/day; chloroquine 250 mg/day; IV cyclophosphamide 500–1000 mg/m^2^	Methylprednisolone 1 g/day; IV cyclophosphamide 750 mg/m^2^; hydroxychloroquine 300 mg/day; prednisone 2 mg/kg/day	Prednisone 21 mg/kg/day; chloroquine 250 mg/day	Mycophenolate mofetil 2 g/day
Sexual activity	Active	Active	Not active	Active	Active
Period between SLE and GCA, months	22	108	1	48	12
Clinical features at GCA	Vulva	Vulva, vagina, anal	Vulva and anal	Vulva	Vulva, vagina, perineum, and anal
HPV type	HPV DNA of oncogenic group and cervix biopsy	HPV 16, HPV DNA of oncogenic group and cervix biopsy	HPV DNA of oncogenic group and HPV 16	HPV DNA of oncogenic group	HPV 6, 11, and 42

*Treatments at GCA*					
SLE therapy	Prednisone 20 mg/day; azathioprine 150 mg/day; chloroquine 250 mg/day	Prednisone 20 mg/day; chloroquine 250 mg/day; azathioprine 150 mg/day	Intravenous cyclophosphamide 750 mg/m^2^; hydroxychloroquine 300 mg/day; prednisone 2 mg/kg/day	Prednisone 5 mg/day; chloroquine 250 mg/day	Mycophenolate mofetil 2 g/day
HPV	LEEP	CO_2_ laser vaporization	Surgical removal	LEEP	Trichloroacetic acid; TLR7 agonist imiquimod 5% cream; podophyllin 2% oil; operation
Recurrence of GCA	Yes	—	—	—	—

♀: female; GCA: giant condyloma acuminata; SLE: systemic lupus erythematosus; LEEP: loop electrosurgical excisional procedure.

## References

[1] Faco M. M. M., Leone C., Campos L. M. A., Febrônio M. V., Marques H. H. S., Silva C. A. (2007). Risk factors associated with the death of patients hospitalized for juvenile systemic lupus erythematosus. *Brazilian Journal of Medical and Biological Research*.

[2] Vet J. N. I., de Boer M. A., van den Akker B. E. W. M. (2008). Prevalence of human papillomavirus in Indonesia: a population-based study in three regions. *British Journal of Cancer*.

[3] Kreuter A., Potthoff A., Brockmeyer N. H. (2010). Anal carcinoma in human immunodeficiency virus-positive men: results of a prospective study from Germany. *British Journal of Dermatology*.

[4] Ganguly N., Waller S., Stasik C. J., Skikne B. S., Ganguly S. (2008). Giant anal condylomatosis after allogeneic bone marrow transplantation: a rare complication of human papilloma virus infection. *Transplant Infectious Disease*.

[5] Daneshpouy M., Socie G., Clavel C. (2001). Human papillomavirus infection and anogenital condyloma in bone marrow transplant recipient. *Transplantation*.

[6] Tam L.-S., Chan P. K. S., Ho S. C. (2011). Risk factors for squamous intraepithelial lesions in systemic lupus erythematosus: a prospective cohort study. *Arthritis Care & Research*.

[7] Santana I. U., Gomes A. D. N., Lyrio L. D., Rios Grassi M. F., Santiago M. B. (2011). Systemic lupus erythematosus, human papillomavirus infection, cervical pre-malignant and malignant lesions: a systematic review. *Clinical Rheumatology*.

[8] Nath R., Mant C., Luxton J. (2007). High risk of human papillomavirus type 16 infections and of development of cervical squamous intraepithelial lesions in systemic lupus erythematosus patients. *Arthritis Care and Research*.

[9] Lube G., Aikawa N. E., Tacla M. (2014). Condyloma acuminatum by human papilloma virus infection in childhood-systemic lupus erythematosus patients. *Acta Reumatológica Portuguesa*.

[10] Costapinto L., Grassi M. F. R., Serravalle K., Travessa A. C. V., Olavarria V. N. O., Santiago M. B. (2012). Giant disseminated condylomatosis in SLE. *Lupus*.

[11] Thornsberry L., English J. C. (2012). Evidence-based treatment and prevention of external genital warts in female pediatric and adolescent patients. *Journal of Pediatric and Adolescent Gynecology*.

[12] Hariri S., Unger E. R., Sternberg M. (2011). Prevalence of genital human papillomavirus among females in the United States, the National Health and Nutrition Examination Survey, 2003–2006. *The Journal of Infectious Diseases*.

[13] Martínez-Martínez M. U., Baranda-Cándido L., Abud-Mendoza C. (2013). Cutaneous papillomavirus infection in patients with rheumatoid arthritis or systemic lupus erythematosus. A case-control study. *Lupus*.

[14] Amador-Molina A., Hernández-Valencia J. F., Lamoyi E., Contreras-Paredes A., Lizano M. (2013). Role of innate immunity against human papillomavirus (HPV) infections and effect of adjuvants in promoting specific immune response. *Viruses*.

[15] Renoux V. M., Bisig B., Langers I. (2011). Human papillomavirus entry into NK cells requires CD16 expression and triggers cytotoxic activity and cytokine secretion. *European Journal of Immunology*.

[16] Henriques A., Teixeira L., Inês L. (2013). NK cells dysfunction in systemic lupus erythematosus: relation to disease activity. *Clinical Rheumatology*.

[17] Mendoza-Pinto C., Garcia-Carrasco M., Vallejo-Ruiz V. (2013). The impact of glucocorticoids and anti-cd20 therapy on cervical human papillomavirus infection risk in women with systemic lupus erythematosus. *Clinics*.

[18] Abud-Mendoza C., Cuevas-Orta E., Santillán-Guerrero E. N. (2013). Decreased blood levels of B lymphocytes and NK cells in patients with systemic lupus erythematosus (SLE) infected with papillomavirus (HPV). *Archives of Dermatological Research*.

[19] Klumb E. M., Pinto A. C., Jesus G. R. (2010). Are women with lupus at higher risk of HPV infection?. *Lupus*.

[20] Katzung B. G. (2006). *Basic and Clinical Pharmacology*.

[21] Maniar K. P., Ronnett B. M., Vang R., Yemelyanova A. (2013). Coexisting High-grade Vulvar Intraepithelial Neoplasia (VIN) and Condyloma Acuminatum: independent lesions due to different HPV types occurring in immunocompromised patients. *American Journal of Surgical Pathology*.

[22] Fairley C. K., Hocking J. S., Gurrin L. C., Chen M. Y., Donovan B., Bradshaw C. S. (2009). Rapid decline in presentations of genital warts after the implementation of a national quadrivalent human papillomavirus vaccination programme for young women. *Sexually Transmitted Infections*.

[23] Mok C. C., Ho L. Y., Fong L. S., To C. H. (2013). Immunogenicity and safety of a quadrivalent human papillomavirus vaccine in patients with systemic lupus erythematosus: a case-control study. *Annals of the Rheumatic Diseases*.

[24] Aubin F., Martin M., Puzenat E. (2011). Genital human *Papillomavirus* infection in patients with autoimmune inflammatory diseases. *Joint Bone Spine*.

